# Empathy among Medical Students: Is There a Relation with Quality of Life and Burnout?

**DOI:** 10.1371/journal.pone.0094133

**Published:** 2014-04-04

**Authors:** Helena B. M. S. Paro, Paulo S. P. Silveira, Bruno Perotta, Silmar Gannam, Sylvia C. Enns, Renata R. B. Giaxa, Rosuita F. Bonito, Mílton A. Martins, Patricia Z. Tempski

**Affiliations:** 1 Department of Obstetrics and Gynecology, Federal University of Uberlândia, Uberlândia, Minas Gerais, Brazil; 2 Department of Public Health, Federal University of Uberlândia, Uberlândia, Minas Gerais, Brazil; 3 Department of Medicine, School of Medicine of the University of São Paulo, São Paulo, São Paulo, Brazil; 4 Department of Pathology, School of Medicine of the University of São Paulo, São Paulo, São Paulo, Brazil; 5 Center for Development of Medical Education, School of Medicine of the University of São Paulo, São Paulo, São Paulo, Brazil; 6 Evangelical Medical School of Paraná, Curitiba, Paraná, Brazil; 7 University of the City of São Paulo, São Paulo, São Paulo, Brazil; 8 University of Fortaleza, Fortaleza, Ceará, Brazil; Universidade de Brasília, Brazil

## Abstract

**Background:**

We aimed to assess medical students' empathy and its associations with gender, stage of medical school, quality of life and burnout.

**Method:**

A cross-sectional, multi-centric (22 medical schools) study that employed online, validated, self-reported questionnaires on empathy (Interpersonal Reactivity Index), quality of life (The World Health Organization Quality of Life Assessment) and burnout (the Maslach Burnout Inventory) in a random sample of medical students.

**Results:**

Out of a total of 1,650 randomly selected students, 1,350 (81.8%) completed all of the questionnaires. Female students exhibited higher dispositional empathic concern and experienced more personal distress than their male counterparts (p<0.05; *d*≥0.5). There were minor differences in the empathic dispositions of students in different stages of their medical training (p<0.05; *f*<0.25). Female students had slightly lower scores for *physical* and *psychological* quality of life than male students (p<0.05; *d*<0.5). Female students scored higher on *emotional exhaustion* and lower on *depersonalization* than male students (p<0.001; *d*<0.5). Students in their final stage of medical school had slightly higher scores for *emotional exhaustion*, *depersonalization* and *personal accomplishment* (p<0.05; *f*<0.25). Gender (β = 0.27; p<0.001) and *perspective taking* (β = 0.30; p<0.001) were significant predictors of *empathic concern* scores. *Depersonalization* was associated with lower *empathic concern* (β = −0.18) and *perspective taking* (β = −0.14) (p<0.001). *Personal accomplishment* was associated with higher *perspective taking* (β = 0.21; p<0.001) and lower *personal distress* (β = −0.26; p<0.001) scores.

**Conclusions:**

Female students had higher empathic concern and personal distress dispositions. The differences in the empathy scores of students in different stages of medical school were small. Among all of the studied variables, *personal accomplishment* held the most important association with decreasing *personal distress* and was also a predicting variable for *perspective taking*.

## Introduction

Empathy is an important component of medical professionalism [1], [2] and has been frequently associated with improvements in the health outcomes and the quality of care in clinical practice [3], [4]. Empathy is the ability to share, understand and respond with care to the experiences of others [5]. Being empathic involves cognitive and emotional reactions, such as actively listening to, identifying and understanding the concerns and emotions of others, and conveying this understanding [3], [6], [7].

Empathy is generally viewed as a relatively stable constitutional trait [7], [8], [9]. In accordance, previous studies demonstrate correlations between empathy, gender [10], [11] and personality [12]–[14]. Supporting the idea of empathy as a constitutional trait, higher empathy scores have been consistently demonstrated among female medical students [10], [11]. Moreover, the association of medical students' empathy and personality traits such as sociability [12], [15], openness to experience and agreeableness [13] has also been confirmed in studies with different methodological approaches.

An alternative view of empathy is that of a mutable state, encompassing both dispositional and constitutional characteristics, in which affective and cognitive responses are sensitive to circumstances [6], [16]. According to this later view, educational experiences have an influence on the development of students' empathy. Studies have shown associations between empathy and situational factors such as year of medical school [17]–[19], [20]–[24]. However, conclusions are contradictory. Most studies conducted in North America conclude that empathy decreases throughout medical school [17]–[19]. Studies held in different parts of the world indicate increases in medical students' empathy under cross-sectional [20]–[22], longitudinal [14] and interventional [22]–[24] designs. We can also find studies showing no variations in empathy level during undergraduate medical training [11], [14], [25].

The well-being or burnout are situational factors that may condition the empathy of students or residents, but such associations remain relatively unexplored [26]–[28]. Some people may be innately more empathic than others. However, considering empathy as a multidimensional construct sensitive to constitutional and situational factors, such an innate empathy may be positively or negatively influenced by individual experiences within or outside the educational environment. Burnout or low perception of quality of life may ‘focus medical students’ attention inward, making it difficult to establish a therapeutic presence for others' [29]–[31]. In accordance, lower empathy scores have been observed among students with burnout or low perceptions of quality of life [26]–[28].

Quality of life may be understood as one's subjective perception of well-being and sense of personal accomplishment within a social and cultural context. It is a multidimensional construct that encompasses at least four dimensions: physical, psychological, social relationships and environment [32]. Previous studies on medical students' quality of life have demonstrated worse perceptions of well-being among females and students in the transition to clinical years [33], [34]. Burnout is defined as an individual response to chronic emotional and interpersonal stressors on the working environment. It involves three dimensions: emotional exhaustion, cynicism and low sense of personal accomplishment [35]. Burnout has been recently studied among medical students and evidence suggests higher levels of exhaustion and depersonalization in the final years of medical education [31], [32], [38]. Students with burnout seem to have less altruistic professional values and to be at higher risk of engaging unprofessional behaviors [39], [40].

Burnout scores were inversely correlated with medical students' empathy scores in a study [28] using the Jefferson Scale of Empathy [12]. In another study using the Interpersonal Reactivity Index (IRI), Thomas et al. also found lower empathy scores among medical students with burnout and with the lowest scores on quality of life [27]. In a study conducted among residents at three institutions, Shanafelt et al. [26] demonstrated lower IRI scores among physicians with lower perceptions of quality of life.

Existing studies have paid little attention to contextual elements in empathy development and examined a restricted number of circumstantial factors that may influence empathy [41]. They also suffer from limitations inherent to convenience sampling and display inherent response bias within only one or a small number of medical schools.

The present study was developed with the goal of understanding the associations between empathy, burnout and well-being of medical students, using a nationally representative sample in Brazil. We assessed empathy among a random sample of medical students from several Brazilian institutions. We aimed to understand the development of medical students' empathy across undergraduate medical education and its association with constitutional variables such as gender as well as with situational factors such as stage of medical school, quality of life and burnout.

We hypothesized that students with better perceptions of quality of life are more attentive to the needs of others, and, thus, are more empathic. We also postulated that burned out students or those with low perception of well-being may exhibit lower levels of empathy.

## Method

This study received approval from the Research Ethics Committee of the **University of Sao Paulo** and all of the local institutional review boards of the participating medical schools prior to data collection: Escola Bahiana de Medicina e Saúde Pública, Faculdade de Medicina de Marília, Faculdade de Medicina de São José do Rio Preto, Faculdade de Ciências Médicas da Paraíba, Faculdade Evangélica do Paraná, Faculdade de Medicina do ABC, Fundação Universidade Federal de Rondônia, Pontifícia Universidade Católica do Rio Grande do Sul, Pontifícia Universidade Católica de São Paulo, Universidade Estadual do Piauí, Universidade Federal do Ceará, Universidade Federal de Ciências da Saúde de Porto Alegre, Universidade Federal de Goiás, Universidade Federal de Mato Grosso do Sul, Universidade Federal do Rio de Janeiro, Universidade Federal do Tocantins, Universidade Federal de Uberlândia, Universidade Estadual Paulista Júlio de Mesquita Filho, Centro Universitário Serra dos Órgãos, Universidade de Fortaleza, Universidade de Passo Fundo.

### Study design and participants

This is a multi-centric study including 22 Brazilian medical schools. Medical schools were chosen according to size, geographic location, public/private status and methodological design (problem-based learning/traditional), in order to represent the diversity of medical schools in Brazil.

The sample size was estimated to demonstrate a medium effect size on major variables at 80% statistical power and 5% maximum type I error [42]. Thus, we calculated that a sample size of 1,152 students would be required to represent the national total of approximately 110,000 Brazilian medical students and facilitate an analysis according to gender and stage of medical training. We then decided to increase the sample to 1,650 students to account for 30% of losses during data collection.

In Brazil, undergraduate medical education lasts six years. Typically, these years are divided into three two-year stages: *initial* (first and second years), *intermediate* (third and fourth years) and *clerkship rotations* (fifth and sixth years). Students from all phases of medical school (at least 10 students from each school year) were selected from a list of computer-generated random numbers. The random selection process was performed in order to obtain an equal proportion of males and females, since the proportion of female students in Brazilian medical schools is about 50%. Students were selected from a list of computer-generated random numbers according to a list of enrolled students provided by each school. Selected students were then invited to participate in the study via e-mail, social networking sites or personal communication at group meetings with local researchers.

### Data collection

Data collection was conducted from August 2011 to August 2012. Selected students were provided with a link to an electronic survey platform. All of the participants provided their written consent to an explanatory letter that appeared on the first page of the survey platform. This letter stated the study goals and guaranteed the confidentiality of the participants. The students had ten days to complete the survey and could participate only once in the study. After completing the survey, the students received automatic feedback on their scores. Additionally, they were provided an opportunity to contact the coordinating researchers for guidance on emotional support if they felt it to be necessary.

### Study measures

All of the participants provided socio-demographic information and answered validated questionnaires on empathy, quality of life and burnout. All of the questionnaires were psychometrically sound [7], [32], [35], [43], [44] and have been used among medical students worldwide. A brief description of these measures is provided below.

#### Interpersonal Reactivity Index

The Interpersonal Reactivity Index (IRI) is a measure of empathy disposition widely used in studies with medical students [10], [11], [26], [27], [45]–[47]. We measured the medical students' empathy on three subscales of the Brazilian validated version of the IRI [48]: (1) *empathic concern*, which “inquires about respondents' feelings of warmth, compassion and concern for others” (emotional empathy domain), (2) *perspective taking*, which “contains items assessing spontaneous attempts to adopt the perspectives of other people and see things from their point of view” (cognitive empathy domain) and (3) *personal distress*, which “measures the personal feelings of anxiety and discomfort that result from observing another's negative experience” [43]. Each subscale comprises seven items and answers are provided on a five-point Likert-like scale (0 = does not describe me well; 4 = describes me very well), with scores ranging from zero to 28. Higher scores in each subscale indicate higher dispositions for empathic concern, perspective taking and personal distress.

We chose to use the IRI due to its advantage of covering both emotive and cognitive dimensions of the empathy construct [43]. By choosing a self-reported questionnaire of empathy disposition, we are aware of the concerns related to its predictive validity regarding the empathic behavior. We know that a high empathy disposition does not necessarily ensure empathic behavior [49]. However, a higher empathy disposition will increase the likelihood that these qualities will be manifested as empathic behavior. Thus, self-reported measures of empathy such as the IRI may be considered a good proxy of the empathic behavior [4].

#### WHOQOL-BREF

The Brazilian version of the World Health Organization Quality of Life Assessment (WHOQOL-BREF) is a generic short-form measure of quality of life that consists of 26 items clustered in four domains: *physical health*, *psychological health*, *social relationships* and *environment*. Answers are provided on a five-point Likert scale, and scores for each domain are transformed into a linear scale that ranges from 0 (least favorable quality of life) to 100 (most favorable quality of life) [32], [50].

#### Maslach Burnout Inventory – Human Services Survey (MBI – HSS)

In order to assess students' self-perception of burnout, we used the Brazilian version of the MBI [51]. This is a standard measure of burnout that includes 22 items scored on a seven-point Likert scale ranging from 0 (never) to 6 (every day). The MBI encompasses three domains: (1) *emotional exhaustion* (score range 0–54), which “assesses feelings of being emotionally overextended and exhausted by one's work”; (2) *depersonalization* (score range 0–30), which “measures an unfeeling and impersonal response towards recipients of one's service, care, treatment or instruction” and (3) *personal accomplishment* (score range 0–48), which “assesses feelings of competence and successful achievement in one's work with people”. Although there are cut-off points that suggest the presence of the burnout syndrome for medical professionals (emotional exhaustion ≥ 27, depersonalization ≥ 10 and personal accomplishment ≤ 33) [44], we considered MBI scores as continuous variables for comparative and regression analysis.

### Statistical analysis

Categorical variables were analyzed with chi-square tests. We used Student's t test to compare major outcome variables between genders and ANOVA to compare scores across students in different stages of medical school. Correlations between variables were analyzed using Pearson's correlation coefficients for continuous variables and Spearman's coefficients for categorical variables. We used forward stepwise multiple regression analysis to evaluate the association of independent variables with all three domains of dispositional empathy. We only included variables with at least moderate correlation coefficients (r>0.3) in the regression models. We set a p value of <0.05 for all statistical analyses. Additionally, we analyzed significant differences through effect-size coefficients. We calculated Cohen's *d* and *f* coefficients (for two- and three-group comparisons, respectively) as well as R^2^ coefficients (for multiple regression analysis). According to Cohen, *d*≥0.5, *f*≥0.25 and R^2^≥0.13 are considered to be moderate values for data interpretation [52].

## Results

Out of a total of 1,650 randomly selected students, 1,350 (81.8%) completed all of the questionnaires on the electronic platform (mean age = 22.76, SD = 3.00, range 17 to 40 years). Losses in the study corresponded to 13 students (0.8%) who did not finish answering the questionnaires on the platform, 13 (0.8) whose data were lost due to technical problems on the web system, and 274 (16.6%) who were unwilling to participate in the study. Of the participating students, 714 were female (52.9%). The respondents were equally distributed across all of the medical school stages: 459 (34.0%) were in the initial stage, 491 (36.4%) were in the intermediate stage and 400 (29.6%) were in clerkship rotations (*X* ^2^ = 0.452; *df* = 2; n = 1,350; p = 0.80).

### Gender differences

Female students had moderately higher *empathic concern* scores than their male counterparts (p<0.001; Cohen's *d* = 0.63). They also scored lower on *personal distress* than male students (p<0.001; Cohen's *d* = 0.35) (**Table 1**, upper panel).

**Table 1:** Dispositional empathy, quality of life and burnout scores according to gender.

		Male (n = 636)	Female (n = 714)	*t*	*df*	p*	Mean dif.	95% CI	Cohen's *d*
		Mean (SD)	Mean (SD)						
	Domains								
**Empathy**	Empathic concern	17.54 (4.51)	20.45 (4.66)	−11.61	1,348	<0.01	−2.91	[−3.40, −2.41]	0.63
	Perspective taking	17.47 (5.11)	17.83 (4.99)	−1.30	1,348	0.19	−0.36	[−0.90, 0,18]	-
	Personal distress	11.46 (3.93)	12.91 (4.36)	−6.39	1,348	<0.01	−1.45	[−1.89, −1.01]	0.35
**Quality of life**	WHOQOL-Physical Health	67.31 (14.38)	63.36 (14.75)	4.97	1,348	<0.01	3.95	[2.39, 5.51]	0.27
	WHOQOL- Psychological	64.16 (15.03)	59.54 (15.96)	5.46	1,348	<0.01	4.62	[2.96, 6.28]	0.30
	WHOQOL-Social Relationships	63.76 (20.32)	63.39 (19.51)	0.34	1,348	0.73	0.37	[−1.76, 2.50]	-
	WHOQOL-Environment	63.97 (14.19)	63.69 (14.00)	0.37	1,348	0.71	0.28	[−1.22, 1.79]	-
**Burnout**	Emotional Exhaustion	25.67 (9.90)	27.68 (9.61)	−3.80	1,348	<0.01	−2.02	[−3.06, −0.98]	0.21
	Depersonali-zation	9.16 (5.90)	8.00 (5.53)	3.72	1,348	<0.01	1.16	[0.55, 1.77]	0.20
	Personal accomplishment	33.93 (7.67)	33.59 (7.56)	0.83	1,348	0.41	0.34	[−0.47, 1.16]	-

*Student's t.

Compared with males, the female students had lower scores on the *physical* (p<0.001; Cohen's *d* = 0.27) and *psychological* (p<0.001; Cohen's *d* = 0.30) domains of quality of life (**Table 1**, middle panel).

We observed different burnout score patterns between genders. Higher scores for *emotional exhaustion* were noted among female students (p<0.001; Cohen's *d* = 0.21), whereas higher scores for *depersonalization* were found among males (p<0.001; Cohen's *d* = 0.20) (**Table 1**, lower panel).

### Differences across stages of medical education

A comparison of dispositional empathy across students in different stages of their medical education revealed subtle differences in *personal distress* scores, with lower scores among males in their clerkship years (p = 0.02; Cohen's *d* = 0.11). *Empathic concern* and *perspective taking* scores did not differ according to stage of medical school (p>0.05) (**Table 2**, upper panel). Among female students, we found lower *perspective taking* (p = 0.01; Cohen's *d* = 0.11) and *personal distress* (p = 0.01; Cohen's *d* = 0.11) scores among students during their clerkship years (**Table 3**, upper panel).

**Table 2:** Male students' empathy, quality of life and burnout scores according to medical school stage.

	Domains	1^st^ and 2^nd^ years	3^rd^ and 4^th^ years	5^th^ and 6^th^ years	F	df	p*	Mean square	95% CI	Cohen's *f*
		Mean (SD)	Mean (SD)	Mean (SD)						
Male		n = 211	n = 232	n = 193						
Empathy	Empathic concern	17.99 (4.67)	17.52 (4.35)	17.08 (4.51)	2.03	633	0.13	20.31	[17.19, 17.89]	-
	Perspective taking	17.48 (5.11)	17.65 (5.08)	17.25 (5.16)	0.32	633	0.73	26.13	[17.08, 17.87]	-
	Personal distress	12.05^a^ (3.91)	11.30^a, b^ (3.90)	11.00^b^ (3.92)	3.91	633	**0.02**	15.30	[11.15, 11.76]	0.11
**Quality of life**	WHOQOL-Physical Health	65.89 (15.08)	67.30 (13.76)	68.87 (14.24)	2.17	633	0.12	206.01	[66.19, 68.43]	-
	WHOQOL- Psychological	64.59 (15.59)	63.94 (13.68)	63.97 (16.00)	0.13	633	0.88	226.58	[62.99, 65.33]	-
	WHOQOL-Social Relationships	64.10 (19.56)	64.19 (20.24)	62.87 (21.28)	0.27	633	0.77	413.74	[62.18, 65.34]	-
	WHOQOL-Environment	62.75 (14.44)	64.12 (14.25)	65.14 (13.79)	1.45	633	0.24	201.04	[62.87, 65.08]	-
**Burnout**	Emotional Exhaustion	24.50^a^ (10.02)	25.48^a, b^ (9.38)	27.16^b^ (10.24)	3.71	633	**0.02**	97.27	[24.89, 26.44]	0.11
	Depersonali-zation	7.79^a^ (5.39)	8.84^a^ (5.66)	11.05^b^ (6.26)	16.74	633	**<0.01**	33.21	[8.70, 9.62]	0.23
	Personal accomplishment	32.82^a^ (8.07)	33.60^a^ (7.90)	35.55^b^ (6.65)	6.82	633	**<0.01**	57.82	[33.34, 34.53]	0.15

*ANOVA. Means followed by equal letters do not differ according to Tukey's post-hoc test.

**Table 3:** Female students' empathy, quality of life and burnout scores according to medical school stage.

	Domains	1^st^ and 2^nd^ years	3^rd^ and 4^th^ years	5^th^ and 6^th^ years	F	*df*	p*	Mean square	95% CI	Cohen's *f*
		Mean (SD)	Mean (SD)	Mean (SD)						
Female		n = 248	n = 259	n = 207						
**Empathy**	Empathic concern	20.98 (4.55)	20.31 (4.52)	19.98 (4.92)	2.81	711	0.06	21.63	[20.11, 20.79]	-
	Perspective taking	18.33^a^ (4.83)	18.01^a, b^ (4.95)	17.01^b^ (5.13)	4.21	711	**0.01**	24.63	[17.47, 18.20]	0.11
	Personal distress	13.28^a^ (4.51)	13.16^a^ (4.23)	12.14^b^ (4.25)	4.66	711	**0.01**	18.78	12.59, 13.23]	0.11
**Quality of life**	WHOQOL-Physical Health	61.77 (15.84)	64.49 (14.67)	63.85 (13.31)	2.34	711	0.10	216.67	[62.28, 64.44]	-
	WHOQOL- Psychological	59.81 (16.09)	60.60 (16.32)	57.89 (15.27)	1.72	711	0.18	254.20	[58.37, 60.71]	-
	WHOQOL-Social Relationships	65.49^a^ (18.32)	63.45^a, b^ (19.97)	60.79^b^ (20.09)	3.30	711	**0.04**	378.23	[61.95, 64.82]	0.10
	WHOQOL-Environment	62.87 (14.75)	65.23 (13.67)	62.76 (13.37)	2.46	711	0.09	195.18	[62.66, 64.72]	-
**Burnout**	Emotional Exhaustion	27.00^a^ (10.06)	26.85^a^ (9.47)	29.55^b^ (8.99)	5.59	711	**<0.01**	91.10	[26.98, 28.39]	0.12
	Depersonali-zation	6.88^a^ (5.24)	7.74^a^ (5.51)	9.68^b^ (5.51)	15.56	711	**<0.01**	29.37	[7.60, 8.41]	0.21
	Personal accomplishment	33.80 (7.76)	32.98 (7.68)	34.10 (7.15)	1.39	711	0.25	57.12	[33.03, 34.15]	-

*ANOVA. Means followed by equal letters do not differ according to Tukey's post-hoc test.

We also observed lower *social relationships* scores among females in their final years of medical school (p = 0.04; Cohen's *d* = 0.10; **Table 3**, middle panel). There were no differences among the other dimensions of quality of life across the phases of medical education (p>0.05; **Tables 2** **and** **3**, middle panel).

Students in their final years of medical school (clerkship students) also had higher *emotional exhaustion* and *depersonalization* scores. This pattern was similar for males (p<0.01; Cohen's *d*≤0.23; **Table 2**, lower panel) and females (p<0.01; Cohen's *d*≤0.21; **Table 3**, lower panel). Male clerkship students also had higher *personal accomplishment* scores than students in their initial and intermediate stages of medical school (p<0.01; Cohen's *d* = 0.15; **Table 2**, lower panel). This difference was not observed among female students (p>0.05; **Table 3**, lower panel).

### Empathy as a function of quality of life and burnout scores

For male students, the *personal distress* scores were inversely correlated with the *psychological* quality of life (*r* = -0.4; p<0.001) and *personal accomplishment* scores (*r* = −0.3; p<0.01; **Table 4**). For female students, the *personal accomplishment* scores were moderately correlated with the *personal distress* (*r* = −0.3; p<0.001) and *perspective taking* scores (*r* = 0.4; p<0.001; **Table 5**).

**Table 4:** Pearson's correlation coefficients among empathy, quality of life and burnout domains (male students).

	Stage†	Age§	EEx§	Dep§	PersA§	EC§	PT§	PD§	WhoPh§	WhoPs§	WhoS§	WhoE§
Stage	1											
Age	0.6**	1										
EEx	0.1**	0.0	1									
Dep	0.2**	0.0	0.6**	1								
PersA	0.1**	0.1**	−0.2**	−0.2**	1							
EC	−0.1*	0.0	0.0	−0.2**	0.2**	1						
PT	0.0	0.0	−0.2**	−0.3**	0.2**	0.4**	1					
PD	−0.1**	−0.1**	0.3**	0.2**	−0.3**	0.2**	−0.1**	1				
WhoPh	0.1*	0.0	−0.5**	−0.3**	0.4**	0.0	0.1*	−0.2**	1			
WhoPs	0.0	0.0	−0.5**	−0.4**	0.5**	0.0	0.2**	−0.3**	0.7**	1		
WhoS	0.0	−0.1*	−0.3**	−0.3**	0.3**	−0.1	0.1**	−0.2**	0.4**	0.6**	1	
WhoE	0.1	−0.1*	−0.3**	−0.3**	0.3**	−0.1	0.1	−0.2**	0.6**	0.5**	0.4**	1

†Spearman coefficients;

§Pearson coefficients;

*p<0.05;

^**^p<0.01.

EEx: Emotional exhaustion; Dep: Depersonalization; PersA: Personal accomplishment; EC: Empathic concern; PT: Perspective taking; PD: Personal distress; WhoPhy: WHOQOL-Physical Health; WhoPs: WHOQOL-Psychological; WhoS: WHOQOL-Social relationships; WhoE: WHOQOL-Environment.

**Table 5:** Pearson's correlation coefficients among empathy, quality of life and burnout domains (female students).

	Stage†	Age§	EEx§	Dep§	PersA§	EC§	PT§	PD§	WhoPh§	WhoPs§	WhoS§	WhoE§
Stage	1											
Age	0.7**	1										
EEx	0.1**	0.0	1									
Dep	0.2**	0.1	0.5**	1								
PersA	0.0	0.1*	−0.3**	−0.3**	1							
EC	−0.1*	0.0	−0.1**	−0.3**	0.3**	1						
PT	−0.1*	0.0	−0.2**	−0.3**	0.4**	0.4**	1					
PD	−0.1**	−0.1**	0.3**	0.1**	−0.3**	0.1	−0.1**	1				
WhoPh	0.1	0.0	−0.5**	−0.3**	0.4**	0.1	0.2**	−0.2**	1			
WhoPs	−0.1	0.0	−0.5**	−0.4**	0.5**	0.1**	0.2**	−0.3**	0.7**	1		
WhoS	−0.1**	0.0	−0.4**	−0.3**	0.4**	0.1	0.2**	−0.2**	0.4**	0.6**	1	
WhoE	0.0	−0.1	−0.4**	−0.2**	0.3**	0.1*	0.1**	−0.1*	0.5**	0.5**	0.4**	1

†Spearman coefficients;

§Pearson coefficients;

*p<0.05;

^**^p<0.01.

EEx: Emotional exhaustion; Dep: Depersonalization; PersA: Personal accomplishment; EC: Empathic concern; PT: Perspective taking; PD: Personal distress; WhoPhy: WHOQOL-Physical Health; WhoPs: WHOQOL-Psychological; WhoS: WHOQOL-Social relationships; WhoE: WHOQOL-Environment.

Overall, the empathy scores were weakly correlated with quality of life (r<0.3) and moderately correlated with burnout. In our sample, scores on *physical*, *mental*, *social* and *environmental* domains of quality of life were correlated with *perspective taking* and *personal distress* scores (r≤0.3; p<0.05). The *empathic concern* scores were inversely correlated with *male gender* (r = -0.3; p<0.001) and *depersonalization* scores (r = −0.3; p<0.001). *Perspective taking* scores were inversely correlated with *depersonalization* scores (r = −0.3; p<0.001) and positively correlated with *personal accomplishment* (r = 0.3; p<0.001) and *empathic concern* (r = 0.4; p<0.001). *Personal distress* scores were inversely correlated with *personal accomplishment* (*r* = −0.3; p<0.001) and positively correlated with *emotional exhaustion* scores (*r* = 0.3; p<0.01). We found weak correlations between age (r≤0.1) or stage of medical education (r<0.3; p<0.05) and dispositional empathy scores (**Table 6**).

**Table 6:** Correlation coefficients among sociodemographic variables, empathy, quality of life and burnout domains (total sample).

	Gender†	Stage†	Age§	EEx§	Dep§	PersA§	EC§	PT§	PD§	WhoPh§	WhoPs§	WhoS§	WhoE§
Gender	1												
Stage	0.0	1											
Age	−0.1	0.6**	1										
EEx	0.1**	0.1**	0.0	1									
Dep	−0.1**	0.2**	−0.1	0.5**	1								
PersA	0.0	0.1*	−0.1**	−0.3**	−0.3**	1							
EC	−0.3**	−0.1**	0.0	0.0	−0.3**	0.2**	1						
PT	0.0	−0.1*	0.0	−0.2**	−0.3**	0.3**	0.4**	1					
PD	0.2**	0.1**	0.1**	0.3**	0.1**	−0.3**	0.2**	−0.1**	1				
WhoPh	−0.1**	0.1*	0.0	−0.5**	−0.3**	0.4**	0.0	0.1**	−0.2**	1			
WhoPs	−0.1**	0.0	0.0	−0.5**	−0.3**	0.5**	0.0	0.2**	−0.3**	0.7**	1		
WhoS	0.0	−0.1*	−0.1*	−0.3**	−0.3**	0.3**	0.0	0.2**	−0.2**	0.4**	0.6**	1	
WhoE	0.0	0.0	−0.1**	−0.3**	−0.3**	0.3**	0.0	0.1*	−0.1**	0.5**	0.5**	0.4**	1

†Spearman coefficients;

§Pearson coefficients;

*p<0.05;

^**^p<0.01.

EEx: Emotional exhaustion; Dep: Depersonalization; PersA: Personal accomplishment; EC: Empathic concern; PT: Perspective taking; PD: Personal distress; WhoPhy: WHOQOL-Physical Health; WhoPs: WHOQOL-Psychological; WhoS: WHOQOL-Social relationships; WhoE: WHOQOL-Environment.

We employed forward stepwise multiple regression analysis to assess the association of the potential predictors of the empathy scores. Independent variables were selected according to the presence of moderate correlations with dispositional empathy scores (r>0.3). Thus, age and stage of medical education were not included in the regression models.

We obtained a model with 24% of variance prediction for medical students' *empathic concern* scores (R^2^ = 0.24; p<0.001; **Table 7**). *Male gender* (β = −0.27; p<0.001) and *perspective taking* scores (β = 0.30; p<0.001) were the most important predictors of this model. In the determination of *perspective taking* scores (R^2^ = 0.20; p<0.001), *empathic concern* (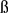 = 0.28; p<0.001) and *personal accomplishment* (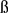 = 0.21; p<0.001) were important predictors. *Personal accomplishment* (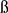 = −0.26; p<0.001) was also an important predictor of lower *personal distress* scores (R^2^ = 0.13; p<0.001) (**Table 7**).

**Table 7:** Multiple linear regression analysis of dispositional empathy domains among medical students.

	Empathic concern						Perspective taking							Personal distress			
*R^2^*	0.24						0.20							0.14			
Adjusted *R^2^*	0.24						0.20							0.14			
p	<0.001						<0.001							<0.001			
Dependent variables	B	SE	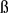	p*	95% CI (B)	B		SE	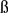	p*	95% CI (B)	B	SE		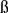	p*	95% CI (B)
(Constant)	16.55	0.52		<0.001	[15.54, 17.57]	8.49		0.82		<0.001	[6.89, 10.09]	16.83	0.80			<0.001	[15.26, 18.40]
Gender§	−2.63	0.23	−**0.27**	<0.001	[−3.09, −2.18]	-		-	-	-	-	-	-		-	-	-
Stage	-	-	-	-	-	-		-	-	-	-	-	-		-	-	-
Age	-	-	-	-	-	-		-	-	-	-	-	-		-	-	-
Empathic concern	N/A	N/A	N/A	N/A	-	0.29		0.03	**0.28**	<0.001	[0.24, 0.34]	-	-		-	-	-
Perspective taking	0.28	0.02	**0.30**	<0.001	[0.24, 0.33]	N/A		N/A	N/A	N/A	N/A	-	-		-	-	-
Personal distress	-	-	-	-	-	-		-	-	-	-	N/A	N/A		N/A	N/A	N/A
Emotional Exhaustion	-	-	-	-	-	-		-	-	-	-	0.06	0.01		**0.14**	<0.001	[0.04, 0.09]
Depersonalization	−0.15	0.02	−**0.18**	<0.001	[−0.19, −0.11]	−0.12		0.02	−**0.14**	<0.001	[−0.17, −0.8]	-	-		-	-	-
Personal accomplishment	-	-	-	-	-	0.14		0.02	**0.21**	<0.001	[0.11, 0.17]	−0.12	0.02		−**0.22**	<0.001	[−0.05, −0.02]
WHOQOL – Physical Health	-	-	-	-	-	-		-	-	-	-	-	-		-	-	-
WHOQOL – Psychological	-	-	-	-	-	-		-	-	-	-	−0.03	0.01		−**0.12**	<0.001	[−0.15, −0.09]
WHOQOL – Social relations	-	-	-	-	-	-		-	-	-	-	-	-		-	-	-
WHOQOL – Environment	-	-	-	-	-	-		-	-	-	-	-	-		-	-	-

^*^ANOVA;

§Gender was coded as: 0 = female; 1 = male; B: unstandardized coefficient; SE: Standard error; 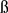: standardized coefficient.

- Independent variable not included in the regression model due to weak/absent correlation with dependent variable

N/A: not applicable.

## Discussion

In this study, we could demonstrate significant inverse associations between empathy and burnout in a large, random sample of medical students. We did not confirm our hypothesis that students with better perceptions of quality of life would report higher empathic dispositions in the relationship with others.

Additionally, we highlighted important gender differences in relation to the variables analyzed in our study. Female students scored higher than their male counterparts in the emotional domains of empathy, but they did not differ in their cognitive scores (perspective taking). Previous studies on empathy that also employed self-reported measures show similar results [10], [11]. It is important to notice that gender differences seem to occur more frequently in studies in which the empathy measures offer respondents situational clues as to the attitude being assessed (measure obviousness) [53]. Empathy scores obtained by self-reported measures may indicate social desirability in the responses. In our study, women were also more emotionally exhausted, and men were more depersonalized. The different coping strategies employed by men and women may explain such gender differences. Our results also indicate that women have lower perceptions of the physical and psychological domains of quality of life than men do. We know that women may report more anxiety, distress and physical symptoms than men [54], [55]. They also have worse perceptions of their academic achievement than male students [56], indicating a more critical self-perception among female students. Moreover, high academic demands in a gender-discriminatory environment during the final years of medical school [57], [58] may result in social isolation and this may explain why scores on perceptions about social relationships are lower among clerkship female students.

Apart from the small difference on the perception of the social domain of quality of life among female students in their final years of medical school, there were no differences in our students' quality of life during the different phases of medical education. Our results are somewhat surprising given existing evidence of lower perceptions of quality of life among students in transition to clinical years of medical education [33], [34]. Optimizing students' quality of life should be a concern of medical educators as recent findings suggest that perceptions of emotional, psychological and social well-being are positively associated with students' altruistic professional values [40]. Our results also suggest that students maintain their empathic disposition throughout medical school. In fact, we found slightly *lower* perspective taking dispositions among female students in their clerkship rotations. Previous studies indicate that a stagnation [11], [14], [25], [27] or even a decline [17]-[19], [40], [41], [59] of emotional and cognitive empathic skills occurs in the course of medical school. However, we argue that the significant differences that indicate a decline in empathy in these studies do not always reflect important changes in practical behavior. Although most studies on medical students' empathy have used validated self-reported questionnaires, the extent to which scores can be translated into the behavioral expression of empathy remains uncertain [16]. Small effect sizes [60] and short-term follow up are also important caveats for interpreting these results.

It is possible that our results of lower scores of empathy among females in the final years of medical school be due to curricular designs with few opportunities to learn and develop empathy or even to the higher levels of distress observed in more advanced years of medical training. Our results confirm higher levels of emotional exhaustion and depersonalization among students in the final years of medical education. This stage of medical training is characterized by high academic overload as a result of clinical rotations and closer contact with patients [36], [37], [61]. Students' higher scores on burnout, particularly on depersonalization, could be a possible explanation for lower dispositional empathy under a theoretical framework in which situational factors also contribute to the construct of empathy.

We were surprised to find clerkship students with higher depersonalization. We believe that depersonalization may result from either the use of passive strategies to cope with stress [62] or insecurity related to the highly competitive selection processes for residency programs. Depersonalized students may experience feelings of cynicism and detachment toward patients [35] and often engage unprofessional behavior [39], [40]. Thus, it is possible to postulate an association between depersonalization and lower dispositions to take the perspective of others, which was further confirmed in the multiple regression analysis.

By using multivariate analysis, we attempted to better understand the correlations among empathy, quality of life and burnout scores. We observed that the model for the empathic concern dispositions was moderately explained by gender. Similarly to previous research [26], [27], we demonstrated significant associations of personal accomplishment and psychological quality of life with diminishing personal distress among students. Personal accomplishment was also a predictor of the perspective taking variance. Correlations among empathy, personal accomplishment and depersonalization scores have been demonstrated in previous studies [27], [28], confirming the hypothesis that the establishment of a truly empathic relationship may also be influenced by either positive or negative situational factors like feelings of personal satisfaction or depersonalization.

The characteristic of impersonal and detached behavior among depersonalized students may explain negative correlations between depersonalization and empathy scores. Because empathy is closely related to broader and stronger social interactions [63], the reason that such negative correlations occur seems clear. Social detachment may be a consequence of the use of passive coping strategies triggered by stressful events [64]. Passive, emotionally driven strategies are also related to lower satisfaction with medical school among students [65], [66].

The personal satisfaction of students was strongly related to higher perspective taking and lower personal distress dispositions. Previous studies have demonstrated that personal accomplishment [63], [66] and professional growth [63] are associated with empathic dispositions among adults and higher satisfaction with care among patients [67]. These results indicate that personal accomplishment and its contributing factors are important variables that medical educators should examine. Certain studies indicate that medical student satisfaction is related to perceptions of medical school as interfering less with social and personal life [66], the presence of strong social ties [68] and efficient social support [69]. Educational strategies should also focus students' feelings of personal accomplishment as a way of enhancing their empathic dispositions.

We must consider several limitations of our study. The use of a self-reported questionnaire for empathic dispositions may not have reflected the real empathic behavior of the students in their professional practice. However, certain authors argue that empathic orientations may result in real behavior [4]. Additionally, some structural issues related to the IRI must be considered. Some authors claim that items of the *personal distress* subscale of the IRI may not be appropriate to assess dispositional empathy among medical students (*eg*, ‘*I tend to lose control during emergencies*’). Excessive distress with the other's situation may be an important characteristic of empathy in the general population, but it may be detrimental for professional performance in the context of health care [15]. However, studies that use empathy scales specific to the medical care context also indicate that the empathic dispositions of medical students are often stunted over the years of their training [11], [14], [25], [27], [59]. Another limitation of our study is related to its cross-sectional design. Longitudinal follow-up will enable causal conclusions to be reached regarding the relations observed in this study. Although hypothesizing empathy as a multidimensional construct encompassing both constitutional and situational variables, our study did not include certain constitutional measures that may also be associated with medical students' empathy. Further studies with a multidimensional approach that includes constitutional traits like coping skills and personality may contribute to elucidate the real association of students' emotional state with their empathic disposition for the other.

Our study also has several important merits. First, with 22 participating medical schools and a random sample of 1,350 students, it is one of the largest multi-centric studies in the medical education literature. Second, the use of a random sample was important for enhancing the generalizability of our results and for mitigating selection bias, a common limitation of studies in medical education research. Third, we could achieve a large national sample of medical students due to the use of an electronic survey platform. The electronic format also enabled students to participate in the study at their convenience and contributed to our low rate of participant losses.

## Conclusions

We conclude that female medical students exhibited higher dispositions for empathic concern and personal distress than their male counterparts. They also displayed lower perceptions of physical and psychological quality of life and higher emotional exhaustion than male students. Such findings have important implications for the psychological well-being of medical students and for the design of training programs on professionalism given the fact that the proportion of female medical students has been increasing internationally.

We observed the presence of burnout in medical students in all stages of medical education and higher emotional exhaustion and depersonalization in the final years of medical school. The empathy of medical students was overall relatively stable across different stages in medical school. Among all of the analyzed variables, personal accomplishment held the most important association with decreasing personal distress and was also a predicting variable for perspective taking. Medical educators must take these findings into account when planning innovative strategies to specifically develop emotional competencies such as empathy during medical school years.
